# PembroWM: A phase II trial to investigate the safety and efficacy of rituximab and pembrolizumab in relapsed/refractory Waldenström's Macroglobulinaemia

**DOI:** 10.1111/bjh.19706

**Published:** 2024-08-19

**Authors:** Jaimal Kothari, Toby Eyre, Ali Rismani, Kushani Ediriwickrema, Darren Edwards, Sevasti Galani, William Wilson, Anthony Lawrie, Laura Clifton‐Hadley, Helen McCarthy, Angela Collins, David Lewis, Suzanne Arulogan, Rebecca Auer, Guy Pratt, Ruth de Tute, Roger Owen, Shirley D'Sa

**Affiliations:** ^1^ Oxford University Hospitals NHS Trust Oxford United Kingdom of Great Britain and Northern Ireland; ^2^ University College Hospital London United Kingdom of Great Britain and Northern Ireland; ^3^ Cancer Research UK and University College London Cancer Trials Centre London United Kingdom of Great Britain and Northern Ireland; ^4^ University Hospitals Dorset NHS Foundation Trust Bournemouth United Kingdom of Great Britain and Northern Ireland; ^5^ Norfolk and Norwich University Hospital NHS Trust Norwich United Kingdom of Great Britain and Northern Ireland; ^6^ Derriford Hospital Plymouth United Kingdom of Great Britain and Northern Ireland; ^7^ Guy's and St Thomas' Hospitals NHS Trust London United Kingdom of Great Britain and Northern Ireland; ^8^ St Bartholomew's Hospital London United Kingdom of Great Britain and Northern Ireland; ^9^ University Hospitals Birmingham NHS Foundation Trust Birmingham United Kingdom of Great Britain and Northern Ireland; ^10^ Leeds Teaching Hospitals NHS Trust Leeds United Kingdom of Great Britain and Northern Ireland; ^11^ Leeds General Infirmary Leeds United Kingdom of Great Britain and Northern Ireland

**Keywords:** clinical trials, haematological malignancy, malignant haematology, therapy, trials, Waldenstrom's Macroglobulinaemia

## Abstract

The optimal therapeutic approach for relapsed/refractory (R/R) Waldenström's Macroglobulinaemia (WM) has not been clearly defined, especially after treatment with chemoimmunotherapy (CIT) and covalent Bruton's tyrosine kinase inhibitors (cBTKi). The PembroWM trial is a multi‐centre, phase II, single‐arm study assessing the safety, tolerability and efficacy of rituximab with pembrolizumab in R/R WM patients who had received at least one prior line of treatment, with all having relapsed post‐CIT and most also exposed to cBTKi. A total of 17 patients were enrolled, with a median age of 70, and median of three prior lines of therapy with 15 either refractory or intolerant of a cBTKi. A significant proportion was identified as genomically high risk with BTKC481, CXCR4 and MYD88 L265P wild‐type aberrations. Twenty‐four‐week overall response rate was 50% (60% CI 39.3%–60.7%), and median duration of response was 11.6 months (IQR: 6.3–17). The median progression‐free survival was 13.6 months (95% CI 3–19.8), and the median overall survival (OS) was not reached. Treatment was well tolerated, with minimal numbers of immune‐mediated AEs typically seen with checkpoint inhibitors. PembroWM is the first study to evaluate the feasibility of PD‐1 axis modulation in WM and has shown that in combination with Rituximab the combination is safe and deliverable.

## Graphical abstract

PembroWM study is a phase II, non‐randomised, single‐arm, open‐label study assessing PD‐1 axis modulation with the combination of pembrolizumab and rituximab in relapsed/refractory Waldenström's Macroglobulinaemia (WM). This combination therapy shows efficacy and tolerability, indicating a viable therapeutic option for heavily pretreated WM patients, including those refractory to covalent BTK inhibitors.

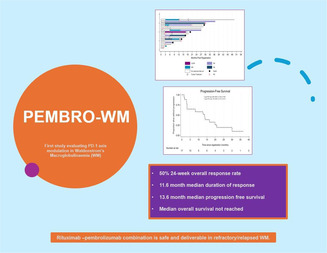

## INTRODUCTION

Waldenström's Macroglobulinaemia (WM) is a low‐grade B‐cell non‐Hodgkin lymphoma, characterised by the presence of a monoclonal immunoglobulin M (IgM) and lymphoplasmacytic lymphoma. It is typically a disease of the elderly with a median age at presentation of over 70 years and an incidence of 0.57 per 100 000 person‐years.^1^, ^2^ *MYD88* L265P activating mutations are seen in over 90% of WM patients, with CXCR4 mutations in approximately 30%. These mutations can be found alone or together and lead to different clinical pictures, outcomes and response to therapies.^3^ WM has a 10‐year overall survival rate of around 70% with improving outcomes, although increasing age is associated with inferior survival mainly driven by non‐lymphoma deaths.^4^, ^5^ Indications for treatment are based on standardised clinical and laboratory criteria.^6^

The cornerstones of WM therapy are rituximab‐based chemoimmunotherapy (CIT) and covalent BTK inhibitors (cBTKi), which can be used either at first‐line or in the relapsed/refractory setting.^7^, ^8^ Data are limited on how best to proceed for patients who relapse or are intolerant to cBTKi after they have also been exposed to CIT. Real‐world data indicate that approximately 20% of WM patients may discontinue ibrutinib within a year for intolerance.^9^ Many patients who are failed by cBTKi treatment can be re‐treated with CIT, and there is encouraging data emerging that non‐covalent reversible BTKi are active post‐cBTKi, but this class of agents is still in clinical trials for WM.^10^, ^11^, ^12^

Soluble PD‐1 ligands have been implicated in T‐cell regulatory function in the WM microenvironment; hence, it is reasonable to postulate that PD‐1 blocking antibodies may be an effective therapeutic intervention in WM.^13^ Pembrolizumab is a potent and highly selective humanised monoclonal antibody of the IgG_4_/kappa isotype, designed to directly block the interaction between PD‐1 and its ligands: PD‐L1 and PD‐L2. PD‐1 inhibition has demonstrated significant efficacy and is a treatment option as monotherapy and in combination with chemotherapy in Hodgkin lymphoma.^14^, ^15^ Single‐agent PD‐1 inhibition with nivolumab in relapsed follicular lymphoma showed very little activity, but data combining pembrolizumab with rituximab in a similar population looked significantly better; hence, there was a logical rationale to investigate the efficacy of PD‐1 blockade in combination with rituximab in relapsed WM.^16^, ^17^

We therefore designed PembroWM, which aimed to assess the ability of pembrolizumab and rituximab (r‐pembro) combination therapy to treat relapsed/refractory WM patients, primarily in cBTKi relapsing/intolerant patients, where there is no accepted standard of care.

## METHODS

### Study design

PembroWM is a phase II, non‐randomised, single‐arm, open‐label UK National Cancer Research Institute study (NCT03630042) aiming to determine the safety, tolerability and efficacy of pembrolizumab in combination with rituximab in relapsed/refractory WM. The study was sponsored by University College London, was conducted in accordance with the declaration of Helsinki and was approved by the West London and GTAC Research and Ethics committee (18/LO/2137), and all participants gave written informed consent prior to participating.

### Inclusion and exclusion criteria

Eligible patients met the WHO WM diagnostic criteria with measurable disease defined as a detectable IgM paraprotein level, had an ECOG performance status (PS) of 0–2, had received one or more prior lines of treatment and required initiation of therapy based on standard international criteria.^1^ Rituximab refractory patients (defined as evidence of progression or relapse on or within 6 months of receiving a rituximab‐containing regimen) were excluded. Patients with a prior history of autoimmune or other forms of haemolysis were also excluded.

### Treatment regimen

Patients were treated on a 3‐week cycle, for a maximum of 18 cycles (1 year) with rituximab 375 mg/m^2^ IV (days 1, 8 and 15 of cycle 1; day 1 of cycles 2, 6, 10 and 18) and pembrolizumab 200 mg IV infusion (day 2 cycle 1; day 1 all other cycles). Plasmapheresis for hyperviscosity was permissible according to local investigator discretion in the first two cycles.

### Assessments

Disease response was assessed with serum paraprotein/immunofixation, bone marrow and CT imaging at 24 weeks and 1 year using the International Working Group for WM response criteria^18^ and serum IgM measurement at 12, 24 and 52 weeks after starting treatment. Quality of life (QOL) was assessed using the QLQ‐30 questionnaire at baseline and at week 24. All adverse events (AEs) that occurred between informed consent and 5 months post last investigational medicinal product (IMP) administration were recorded using CTCAE V.5.

### Endpoints

The primary end‐point was the overall response rate (ORR), defined as complete response (CR), very good partial response (VGPR), partial response (PR) or minor response (MR) at 24 weeks after commencing treatment with r‐pembro. Secondary end‐points included safety and tolerability of the combination of r‐pembro, best response and time to best response, time to next treatment (TTNT), progression‐free survival (PFS), overall survival (OS) and change in QOL between baseline and week 24.

### Statistical analysis

The sample size was calculated using an A'Hern phase II trial design. ORR was hypothesised at 60%, but we aimed to rule out a rate of under 40%. The original design for 80% power required 42 patients based on a one‐sided 5% alpha. However, this was revised to 17 patients on 18 February 2022 (one‐sided 20% alpha) following slower than anticipated recruitment, with a minimum of 9/17 responding patients required. Time to best response was calculated from date of registration until date of best response. TTNT was measured from date of registration to the date of the start of the next line of therapy, and patients who had not started a further line of treatment were censored at the date last seen or date of death. PFS and OS times were calculated from the date of registration to the date of the first event (progression/death or death respectively). All time–to‐event endpoints were analysed using Kaplan–Meier survival analysis. Differences in QOL scores were compared using signed‐rank tests. All analyses were performed in STATA version 18.0 (STATACORP, Texas).

## RESULTS

Seventeen patients were recruited across six sites between October 2019 and February 2022. Table 1 details patient's baseline characteristics; median age was 70 years (IQR: 65–76, range: 44–84), 14 (82%) were male, 16 (94%) had an ECOG PS of 0–1, and the median number of lines of previous treatment was three (IQR 2–4). Fifteen (88%) received a cBTKi as previous treatment prior to enrollment, with 12 (71%) demonstrating cBTKi refractoriness. One patient had received a prior autologous stem cell transplant. Seven (41%) patients were MYD88 L265P mutated including one who also had a CXCR4 mutation, and three patients (18%) had mutations in BTKC481. Four patients had either missing or inadequate samples so no mutational analysis was possible. Six patients had no detectable mutations of any kind. Median IgM levels were 40 g/L (IQR 9.1–57.4 g/L), median bone marrow WM infiltration was 40% (IQR: 20–50) and one (6%) patient exhibited extranodal disease. Eight (47%) experienced symptoms of hyperviscosity and four (50%) of those eight patients required plasmapheresis in cycle 1 or 2.

**TABLE 1:** PembroWM patients’ baseline characteristics.

Baseline characteristics	
Age (years), median (IQR)	70.4 (64.5–76.1)
Range	44–84
Sex, *N* (%)	
Female	3 (17.6%)
Male	14 (82.4%)
ECOG status, *N* (%)	
0	9 (52.9%)
1	7 (41.2%)
2	1 (5.9%)
Previous Rituximab (at any point)	
Yes	17 (100%)
Previous cBTKi (at any point)	
Yes	15 (88.2%)
No	2 (11.8%)
cBTKi as last treatment	
Yes	13 (76.5%)
No	4 (23.5%)
Refractory to cBTK inhibitor, *N* (%)	
Yes	12 (52.9%)
No	5 (47.1%)
Reason for cBTK failure for those who received cBTK, *N* (%)	
Progression	11 (73.3%)
Intolerance due to AEs	3 (20%)
No response	1 (6.7%)
Number of lines of previous WM treatment, median (IQR)	3.0 (2.0–4.0)
Range	1.0–6.0
Bone marrow trephine percentage, median (IQR)	40.0 (20.0–50.0)
Range	10.0–90.0
Prior autologous stem cell transplant, *N* (%)	
Yes	1 (5.9%)
No	16 (94.1%)
IgM (g/L), median (IQR)	39.7 (9.1–57.4)
Range	2.5–71.6
B symptoms, *N* (%)	
Any	5 (29.4%)
Fever	1 (5.9%)
Night sweats	5 (29.4%)
Weight loss	2 (11.8%)
Neuropathy, *N* (%)	1 (5.9%)
Hyperviscosity symptoms, *N* (%)	
Any	8 (47.1%)
Fatigue	8 (47.1%)
Mucosal bleeding	1 (5.9%)
Splenomegaly, *N* (%)	2 (11.8%)
Lymphadenopathy, *N* (%)	4 (23.5%)

Abbreviations: AE, adverse events; cBTKi, covalent BTK inhibitor; IQR, interquartile range.

All patients started treatment, and the median duration was eight cycles (IQR: 4–18, range: 1–18) with five patients (29%) completing all 18 cycles. Six patients stopped the treatment early due to progression (one after three cycles, three after four cycles and two after six cycles). Three withdrew due to adverse events, two due to infection and one hyperhidrosis. Two patients withdrew consent, and one patient stopped because they no longer derived benefit, as per clinician decision (after six cycles). Ten (59%) patients experienced at least one delay to treatment due to AEs (*N* = 7), patient choice (*N* = 1), COVID‐19 restrictions (*N* = 2) or daycare booking pressure (*N* = 2). Two patients received incomplete rituximab doses due to infusion reactions.

**FIGURE 1:**
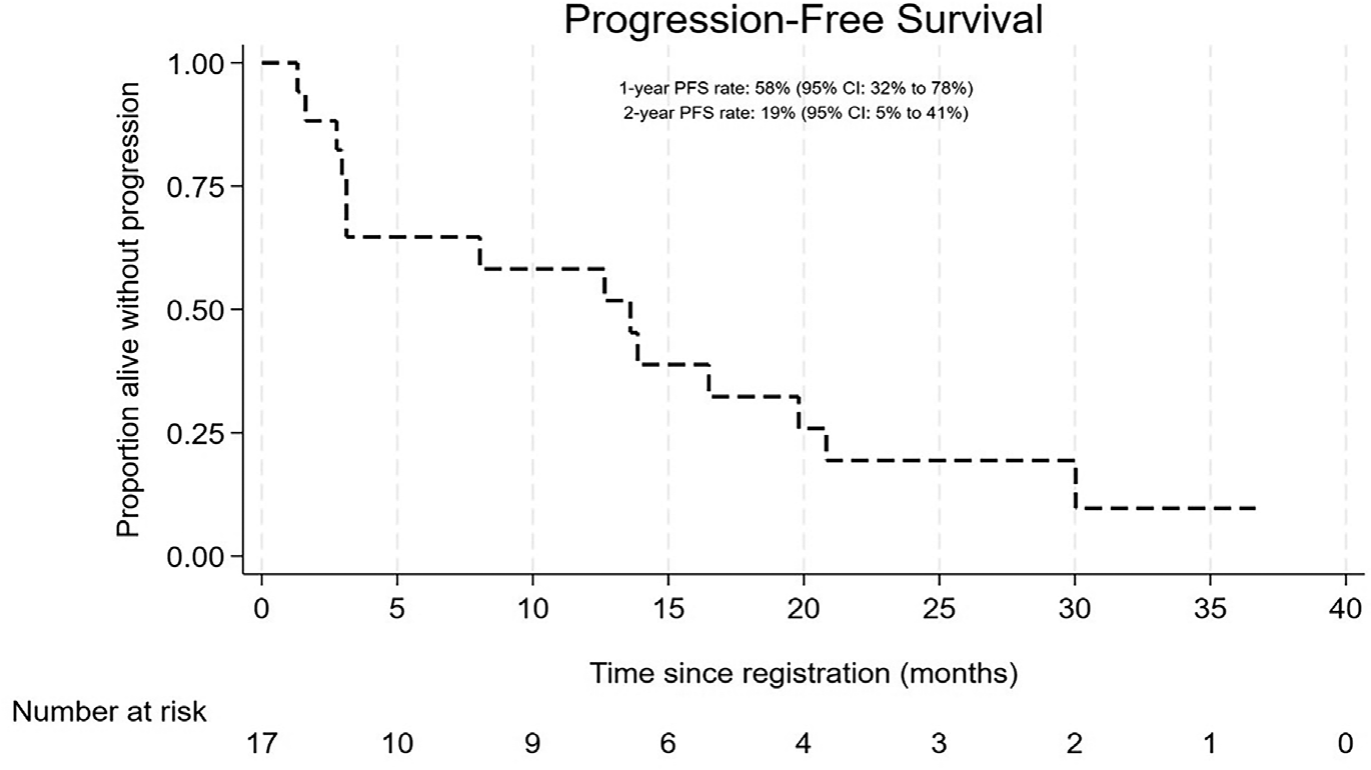
Kaplan–Meier curve displaying progression‐free survival (PFS). The median PFS is 13.6 months (95% CI 3–19.8). 1‐year PFS rate of 58.2% (95% CI 31.7%–77.5%) and 2‐year PFS rate of 19.4% (95% CI 4.8%–41.3%).

**FIGURE 2:**
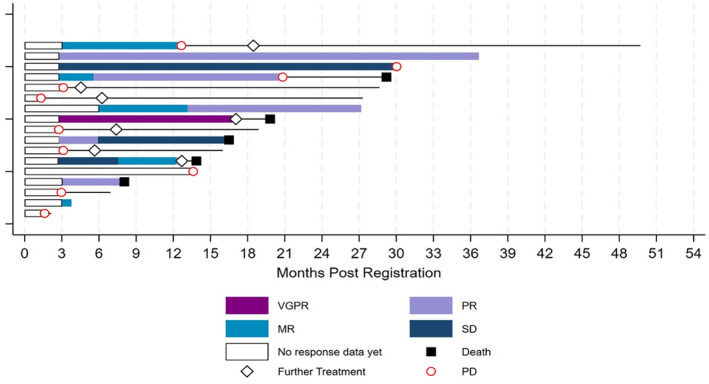
Swimmer's plot of responses/outcomes illustrating international working group for WM criteria of response and duration for all 17 patients. MR, minor response; PD, progressive disease; PR, partial response; SD, stable disease; VGPR, very good partial response.

ORR at 24 weeks was 47.1% (60% CI 36.9%–57.5%) with one patient attaining VGPR, three (18%) PR and four (24%) MR (including one patient who withdrew at week 15 but who had achieved an MR at week 12). One patient withdrew due to toxicity at cycle 1 and was not assessable for response at any time point, excluding this patient who gave an ORR at 24 weeks of 50% (60% CI 39.3%–60.7%). The median time to best response was 3.1 months (IQR: 2.8–5.6) with one additional patient responding at week 12 but showing SD at week 24, giving a best overall response rate of 52.9% (60% CI 40%–65.5%). Six responders progressed or died with median duration of response of 11.6 months (IQR: 6.3–17, range: 5–18 months). With a median follow‐up of 27.3 months (IQR 16–30), there have been 14 PFS events; ten progressions, three deaths without progression and one death from WM with date of progression unknown due to patient withdrawal. The median PFS is 13.6 months (95% CI 3–19.8) with 1‐ and 2‐year PFS rates of 58.2% (95% CI 31.7%–77.5%) and 19.4% (95% CI 4.8%–41.3%) respectively (Figure 1). A Swimmers plot (Figure 2) further depicts responses and outcomes.

**FIGURE 3:**
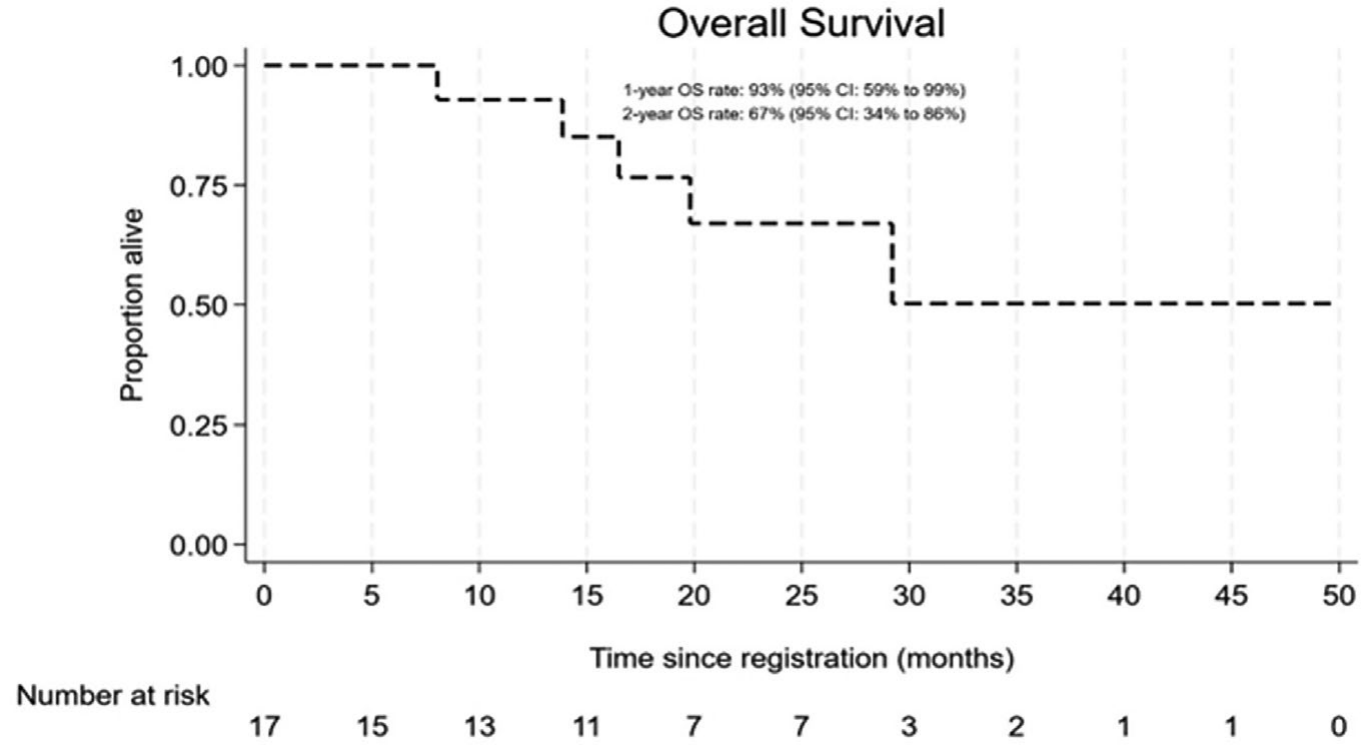
Kaplan–Meier curve illustrating overall survival (OS). Median OS was not reached. 1‐year OS rate of 92.9% (95% CI 59.1%–99%) and 2‐year OS rate of 67% (33.5%–86.4%).

**FIGURE 4:**
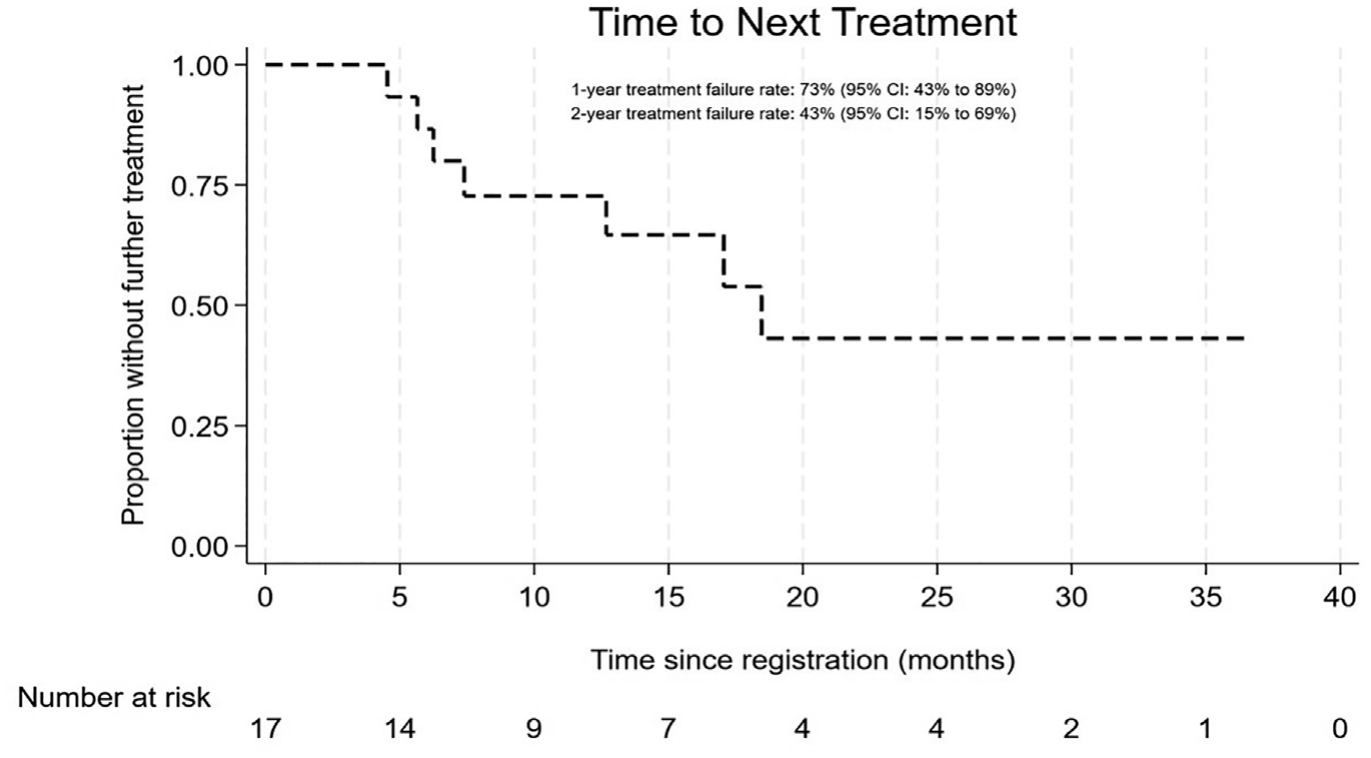
Median TTNT (time to next treatment) of 18.5 months (95% CI 6.2‐NR). 1‐year TTNT of 72.7% (95% CI 42.5%–88.8%) and 2‐year TTNT rate of 43.1% (14.9%–68.9%).

Five patients died; one death was directly attributed to WM, with two due to infections (including one COVID‐19), one due to metastatic melanoma and one due to transplant complications after a subsequent allograft for WM. No deaths were believed to be related to trial treatment. Median OS was not reached, and the 1‐ and 2‐year OS rates were 92.9% (95% CI 59.1%–99%) and 67% (33.5%–86.4%) respectively (Figure 3). Seven patients reported a further line of treatment and the 1‐ and 2‐year time to next treatment (TTNT) rates were 72.7% (95% CI 42.5%–88.8%) and 43.1% (14.9%–68.9%) with a median TTNT of 18.5 months (95% CI 6.2‐NR; Figure 4). Details of subsequent treatments are the following; five patients were given rituximab combination chemotherapy, two with bendamustine, two with bortezomib +/− dexamethasone and one with ixazomib and dexamethasone. A single patient received a rituximab‐free chemotherapy regime consisting of bortezomib, cyclophosphamide and dexamethasone (BCD). One patient had a sibling donor allogeneic transplant. Two patients reported two subsequent lines of treatment, one given BCD and the second given pirtobrutinib.

**FIGURE 5:**
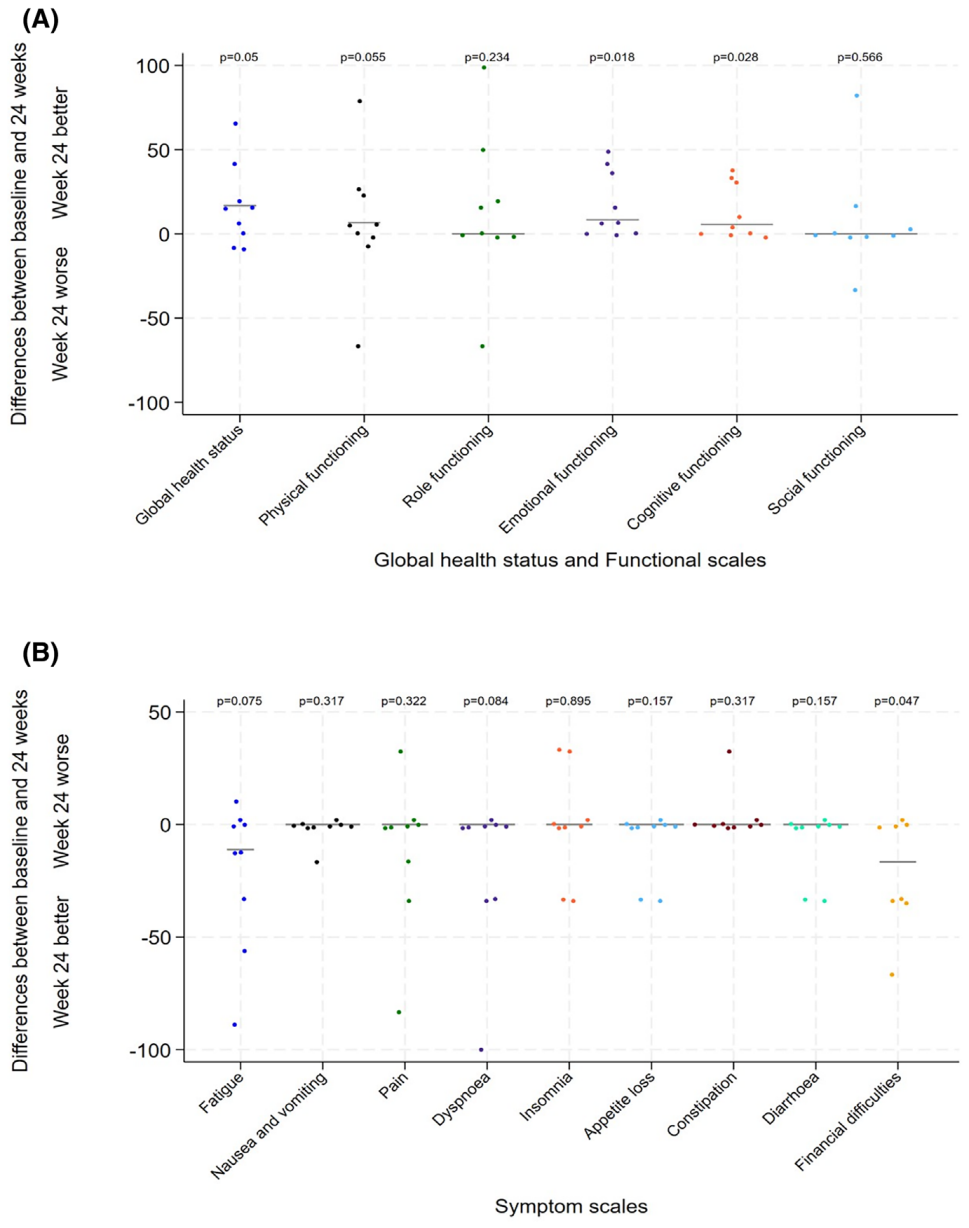
Graphs detailing QLQ‐30 questionnaire differences at baseline compared to week 24. (A) QOL data detailing differences in global health status and functional scales. (B) QOL data detailing differences in symptom scales. Emotional functioning (median difference + 8.3, IQR 0 to 33.3, *p*‐value = 0.02) and cognitive functioning scores (+5.6, IQR 0 to 27.8, *p*‐value = 0.03) demonstrated a significant improvement. Other categories in all scores or symptom scales demonstrated no difference, or an improvement, from baseline to week 24.

AEs occurred in 16 patients (94%). The most common events were anaemia, fever, fatigue, raised creatinine, infusion‐related reactions, dizziness, cough, hypotension and neutropenia, though most were at grades 1–2 only. Thirteen (77%) reported grade 3 or higher adverse events with high frequency of grade 3 infection (29%), including COVID‐19. Three patients (18%) experienced grade 4 AEs; neutropenia, thrombocytopenia and an embolic stroke, which was not related to treatment. One patient experienced grade 5 respiratory failure due to infection, which was also felt to be unrelated to treatment. QOL data were available for nine patients at both baseline and 24 weeks. The majority of patients showed no difference, or an improvement, in all scores and symptom scales from baseline to week 24 (Figure 5); however, only emotional functioning (median difference + 8.3, IQR 0 to 33.3, *p*‐value = 0.02) and cognitive functioning scores (+5.6, IQR 0 to 27.8, *p*‐value = 0.03) demonstrated a significant improvement.

## DISCUSSION

The PembroWM study highlights that combining rituximab and pembrolizumab is feasible, with a reassuring safety profile and offers some disease control in a heavily pretreated population of WM patients. This is the first study to evaluate the efficacy and tolerability of PD‐1 inhibition in WM. It was decided to combine pembrolizumab with rituximab as rituximab is a well‐established therapeutic backbone in relapsed/refractory WM with a predictable safety profile. Other studies in relapsed/refractory B‐NHL have also been set up using this combination, and some of these studies have now been fully reported.^17^

All patients in this study had been exposed to rituximab‐containing CIT, and the majority of patients were either intolerant or refractory to a cBTKi. Standard of care in this relapsed/refractory cohort post‐CIT and cBTKi is yet to be defined with no formally licensed and reimbursed therapies in this specific space. A number of patients refractory to cBTKi had also developed the well‐described BTKC481 mutation.^19^ Other patients were also genomically high risk, with CXCR4 mutations and some being MYD88 L265P wild type. Despite these high‐risk characteristics, just under half of the patients responded to treatment at 24 weeks and had a maximal ORR and a 1‐year PFS rate of over 50%. However, the primary endpoint was not met, as only seven patients responded at week 24. OS was greater than 90% at 1 year, and 67% at 2 years, which is encouraging despite median PFS being around 12 months. This perhaps partly reflects the lack of toxicity of this combination and the ability of these patients to respond further to different CIT and proteosome inhibitor combinations in the relapsed/refractory setting. It is unclear whether the PD1 inhibitor may have contributed to future responses as is hypothesised in other haematological malignancy, for example, Hodgkin lymphoma where PD1 may chemosensitise patients, improving the response to later lines.

The addition of rituximab to a PD‐1 antibody did not lead to significant toxicity or intolerability. The majority of adverse events seen were cytopenias/infections of <grade 3, and there was only one immune‐related AE consistent of the type well described with PD1 inhibitors. Absolute numbers of AEs were low. The serious adverse events related to infection, including one death, were not attributed to trial therapy, and even though most of the recruitment occurred in the early phases of the pandemic, there was no undue excess of COVID‐19. Of note, no cases of haemolysis were seen in this study (patients with history of prior haemolytic anaemia were excluded), and this has been previously reported in treating patients with WM with a PD‐1 antibody.^20^ The tolerability of treatment was further evidenced by QOL data, which showed that on the whole patients did not have a decrease in functional or symptom scale scores following treatment, with a significant benefit seen for emotional and cognitive functioning.

The recruitment challenges experienced during the study led to the sample size being reduced, making it harder to formally evaluate efficacy. Accrual challenges were directly caused by three major factors: (1) reduction in research activity during the initial phases of the COVID‐19 pandemic, (2) the difficulties of recruiting to an investigator‐led study in a rare indication when only a small number of centres can open and (3) the timing of availability and effectiveness of BTK inhibition in WM in the United Kingdom. As ibrutinib had only become available around 24 months prior to the initiation of the study, a significant proportion of patients for whom the study was essentially set up for, were well established and in good remission on ibrutinib. Over the last 18–24 months, the number of patients refractory to cBTKis has increased significantly and it is likely, with time, that studies in the post‐covalent BTKi space will become increasingly feasible.

How to treat patients in the cBTKi refractory setting is becoming increasingly defined; it is clear that non‐covalent BTKi has efficacy,^10^ and patients still may respond to CIT.^21^ Novel agents including bcl‐2 inhibitors are also likely to play an important role.^22^ Given the efficacy results of this study and the way in which the field is moving, it is unlikely that the combination of PD‐1 axis inhibition with rituximab will be further developed. Although not a direct comparison, it is possible that the difference between the r‐pembro combination response rate and PFS and that achievable with rituximab monotherapy in a relapsed/refractory WM population may be minimal.^11^

However, the reduction of the sample size may have meant that a true efficacy signal has been missed. It is also now becoming evident that a number of immune checkpoints are implicated in the WM microenvironment, and this, together with different, potentially profound levels of T‐cell depletion in this multiply treated relapsed/refractory cohort may have led to limited efficacy and this approach may be of more benefit, in earlier lines, or perhaps with a different partner than rituximab.^23^ Given the reassuring safety profile of this combination, it would potentially be interesting to look at combining PD‐1 inhibitors with small molecules (e.g. cBTKi, non‐covalent BTKi or bcl‐2 inhibitors), or with CIT.

Nevertheless, this study has shown that the combination is feasible, and the addition of pembrolizumab to rituximab adds minimal toxicity. It is also possible that modulation of the PD‐1 axis may improve response to further CIT in later lines by combatting t‐cell exhaustion.

As cellular therapeutics evolve in the treatment of haematological malignancy, PD‐1 axis attenuation is likely to continue to be an important avenue to explore, by optimising t‐cell mediated tumour response. This study clearly highlights that modulation of this axis in WM is safe and implementable.

## AUTHOR CONTRIBUTIONS

JK, TE, DE, WW and SD contributed to the study design/methodology. JK and LC‐H contributed to funding and resource acquisition. JK, AR, HM, AC, DL and SD participated in the investigation and/or data collection. RdT and RO co‐ordinated and ran central laboratory analysis. DE, SG, WW and AL contributed to data analysis and/or interpretation. DE, SG, WW and AL verified all data and provided project administration and supervision. All authors contributed to the writing and/or critical revision of the manuscript, approved the manuscript for publication and confirmed access to the primary clinical trial data.

## FUNDING INFORMATION

Supported in part by a research grant from Investigator‐Initiated Studies Program of Merck Sharp & Dohme Limited. The opinions expressed in this paper are those of the authors and do not necessarily represent those of Merck Sharp & Dohme Limited. The study protocol was approved by an NHS Research Ethics Committee (REC reference: 18/LO/2137). The study protocol was also approved by the Medicines and Healthcare products Regulatory Agency. Written informed consent was obtained from all the participants.

## CONFLICT OF INTEREST STATEMENT

No conflicts of interest to disclose.

## Supporting information


Data S1.

## Data Availability

For original data, please contact jaimal.kothari@ouh.nhs.uk.
