# Supplementary material for: PembroWM: A phase II trial to investigate the safety and efficacy of rituximab and pembrolizumab in relapsed/refractory Waldenström's Macroglobulinaemia
Source: Br J Haematol. 2024 Aug 19;205(6):2273–81. doi: 10.1111/bjh.19706 (PMC11637722; doi:10.1111/bjh.19706)
Supplement: Supplementary file 1 — Data S1. [file BJH-205-2273-s001.docx]

**Supplemental Table 1:Adverse Events**

| N=17 | | |
| --- | --- | --- |
| CTCAE v5 | Worst Grade Reported | |
|  | Grade 1-2 | Grade 3+ |
| Any Adverse Event | 3 (17.6%) | 13 (76.5%) |
| **Blood and lymphatic system disorders** | **7 (41.2%)** | **3 (17.6%)** |
| Anemia | 6 (35.3%) | 1 (5.9%) |
| Other/folate deficiency | 1 (5.9%) | . |
| Other/neutropenia | . | 2 (11.8%) |
| **Cardiac disorders** | **6 (35.3%)** | **1 (5.9%)** |
| Atrial fibrillation | . | 1 (5.9%) |
| Sinus tachycardia | 1 (5.9%) | . |
| Atrioventricular block first degree | 1 (5.9%) | . |
| Palpitations | 1 (5.9%) | . |
| Ventricular arrhythmia | 2 (11.8%) | . |
| Other/heart failure with preserved ejection fraction | 1 (5.9%) | . |
| **Ear and labyrinth disorders** | **1 (5.9%)** | **.** |
| Tinnitus | 1 (5.9%) | . |
| **Endocrine disorders** | **1 (5.9%)** | **.** |
| Adrenal insufficiency | 1 (5.9%) | . |
| **Eye disorders** | **2 (11.8%)** | **.** |
| Blurred vision | 2 (11.8%) | . |
| **Gastrointestinal disorders** | **6 (35.3%)** | **.** |
| Dry mouth | 2 (11.8%) | . |
| Hemorrhoids | 2 (11.8%) | . |
| Constipation | 2 (11.8%) | . |
| Diarrhea | 2 (11.8%) | . |
| Nausea | 2 (11.8%) | . |
| Abdominal pain | 2 (11.8%) | . |
| Dyspepsia | 1 (5.9%) | . |
| Other/loose stools | 1 (5.9%) | . |
| Other/mouth ulcer | 1 (5.9%) | . |
| **General disorders and administration site conditions** | **11 (64.7%)** | **.** |
| Chills | 2 (11.8%) | . |
| Localized edema | 2 (11.8%) | . |
| Fever | 6 (35.3%) | . |
| Facial pain | 1 (5.9%) | . |
| Fatigue | 5 (29.4%) | . |
| Other/shoulder pain | 1 (5.9%) | . |
| Other/intermittent pain in fingers | 1 (5.9%) | . |
| **Infections and infestations** | **5 (29.4%)** | **5 (29.4%)** |
| Lymph gland infection | 1 (5.9%) | . |
| Lung infection | 1 (5.9%) | . |
| Thrush | 1 (5.9%) | . |
| Other/infection | . | 2 (11.8%) |
| Other/covid-19 | 1 (5.9%) | 2 (11.8%) |
| Other/chest infection | 1 (5.9%) | . |
| Other/infection (unknown origin) | . | 2 (11.8%) |
| **Injury, poisoning and procedural complications** | **6 (35.3%)** | **2 (11.8%)** |
| Vaccination complication | 1 (5.9%) | . |
| Infusion related reaction | 5 (29.4%) | 2 (11.8%) |
| **Investigations** | **7 (41.2%)** | **5 (29.4%)** |
| Platelet count decreased | . | 2 (11.8%) |
| Weight loss | 1 (5.9%) | . |
| Creatinine increased | 5 (29.4%) | . |
| White blood cell decreased | 3 (17.6%) | 1 (5.9%) |
| Blood bicarbonate decreased | 1 (5.9%) | . |
| Aspartate aminotransferase increased | 2 (11.8%) | . |
| Alanine aminotransferase increased | 2 (11.8%) | . |
| Lymphocyte count decreased | 3 (17.6%) | 1 (5.9%) |
| Serum amylase increased | 1 (5.9%) | . |
| Neutrophil count decreased | 3 (17.6%) | 2 (11.8%) |
| Thyroid stimulating hormone increased | 2 (11.8%) | . |
| Other/white blood cell increased | 2 (11.8%) | . |
| Other/platelet count increased | 1 (5.9%) | . |
| Other/increased neutrophils | 1 (5.9%) | . |
| **Metabolism and nutrition disorders** | **5 (29.4%)** | **1 (5.9%)** |
| Hypercalcemia | 1 (5.9%) | . |
| Hyperuricemia | 2 (11.8%) | . |
| Hypoalbuminemia | 1 (5.9%) | . |
| Hyperkalemia | 1 (5.9%) | . |
| Hypocalcemia | 1 (5.9%) | . |
| Hyponatremia | 2 (11.8%) | 1 (5.9%) |
| Other/iron deficiency | 2 (11.8%) | . |
| **Musculoskeletal and connective tissue disorders** | **5 (29.4%)** | **.** |
| Pain in extremity | 1 (5.9%) | . |
| Muscle cramp | 1 (5.9%) | . |
| Myalgia | 1 (5.9%) | . |
| Flank pain | 1 (5.9%) | . |
| Back pain | 1 (5.9%) | . |
| Other/polymylagia rheumatica | 1 (5.9%) | . |
| **Neoplasms benign, malignant and unspecified (incl cysts and polyps)** | **1 (5.9%)** | **.** |
| Other/excision of basal cell carcinoma | 1 (5.9%) | . |
| Other/malignant melanoma trunk | 1 (5.9%) | . |
| **Nervous system disorders** | **8 (47.1%)** | **2 (11.8%)** |
| Dizziness | 4 (23.5%) | . |
| Headache | 3 (17.6%) | . |
| Paresthesia | 2 (11.8%) | . |
| Stroke | . | 1 (5.9%) |
| Memory impairment | 1 (5.9%) | . |
| Syncope | . | 1 (5.9%) |
| Other/peripheral neuropathy | 2 (11.8%) | . |
| Other/sciatica pain (right leg) | 1 (5.9%) | . |
| **Renal and urinary disorders** | **4 (23.5%)** | **.** |
| Urinary frequency | 1 (5.9%) | . |
| Dysuria | 1 (5.9%) | . |
| Chronic kidney disease | 2 (11.8%) | . |
| **Respiratory, thoracic and mediastinal disorders** | **7 (41.2%)** | **3 (17.6%)** |
| Pleural effusion | 2 (11.8%) | . |
| Cough | 4 (23.5%) | . |
| Dyspnea | 1 (5.9%) | 1 (5.9%) |
| Respiratory failure | . | 1 (5.9%) |
| Stridor | . | 1 (5.9%) |
| Other/hemoptysis | 1 (5.9%) | . |
| **Skin and subcutaneous tissue disorders** | **5 (29.4%)** | **1 (5.9%)** |
| Pruritus | 1 (5.9%) | . |
| Hyperhidrosis | 3 (17.6%) | . |
| Rash acneiform | 3 (17.6%) | . |
| Rash maculo-papular | 1 (5.9%) | 1 (5.9%) |
| **Vascular disorders** | **5 (29.4%)** | **.** |
| Hypotension | 4 (23.5%) | . |
| Flushing | 1 (5.9%) | . |

*1 patient had syncope and dyspnea, but the grade and dates of onset/resolution were not reported, so these are not included in the table. All 17 eligible patients are known to have started treatment. Adverse Events that occurred before treatment and did not occur at a higher grade at later Cycles were not reported. None of the patients reported second primary malignancy. Worst grade seen of each event, i.e. the number and percentage of patients who experience a grade 1-2 or 3+ event at any time point after treatment
